# Pandemic (H1N1) 2009 Infection in Swine Herds, Manitoba, Canada

**DOI:** 10.3201/eid1604.091636

**Published:** 2010-04

**Authors:** Tim Pasma, Tomy Joseph

**Affiliations:** Manitoba Agriculture, Food and Rural Initiatives, Winnipeg, Manitoba, Canada

**Keywords:** Disease transmission, swine, pandemic (H1N1) 2009, virus shedding, zoonoses, viruses, influenza, Canada, expedited, dispatch

## Abstract

In Manitoba, Canada, several swine herds were infected by pandemic (H1N1) 2009 virus in the summer of 2009. Results of several investigations concluded that outbreaks of infection with this virus are similar in duration to outbreaks of infections with swine influenza viruses A (H1N1) and A (H3N2).

On April 21, 2009, the US Centers for Disease Control and Prevention announced the finding of a novel strain of influenza virus A (H1N1), now known as pandemic (H1N1) 2009 virus, in 2 children in southern California (*1*). By June 11, this virus had spread so quickly and extensively among humans that the World Health Organization declared a phase 6 pandemic (*2*). The disease in humans is a self-limiting, uncomplicated respiratory illness with fever; however, severe disease and deaths have occurred (*3*). Clinical signs in humans are generally mild and include fever, slight cough, sneezing, and nasal discharge. Vomiting and diarrhea also have been reported in up to 38% of cases (*3*).

Pandemic (H1N1) 2009 virus also has affected swine. On May 2, 2009, the virus was isolated from a swine herd in Alberta, Canada (*4*). The disease in swine has been reported as mild in field and experimental conditions. Clinical signs in pigs include fever, slight cough, sneezing, and nasal discharge. Diarrhea was also reported in experimentally infected pigs; however, this symptom may have been secondary to the influenza infection (*5*). In experimental infection of pigs, clinical signs peaked on days 4–5 postinfection (*5*).

In Manitoba, Canada, pandemic (H1N1) 2009 virus was first detected in a swine herd on June 30, 2009. During the following months, more outbreaks in Manitoba were reported in farrowing, nursery, and finishing herds. Our study aimed to determine the length of time that virus was shed in swine herds after a field outbreak of pandemic (H1N1) 2009.

## The Study

We studied 5 herds in which pandemic (H1N1) 2009 was diagnosed. We collected information about the production type and number of animals housed in the barn, influenza vaccination status of the herd, date of influenza-like illness in any barn employees before the outbreak, date of the outbreak as determined by onset of clinical signs, and sampling date and number of positive swabs. In each herd, 32 nasal swabs were taken from randomly selected pigs as soon as possible after diagnosis. The procedure was repeated every 7 days until all the samples tested showed negative results.

Nasal swabbing was performed by using a polyester swab with a plastic handle that was placed in a viral transport medium (Starswab Multitrans Collection & Transport System; Starplex Scientific Inc, Etobicoke, Ontario, Canada). The swabs were refrigerated and submitted to the Veterinary Services Diagnostic Laboratory at Manitoba Agriculture, Food and Rural Initiatives (Winnipeg, Manitoba, Canada). Samples were tested by using a generic real-time PCR specific for the genomic RNA segment 7 (matrix gene) of the influenza A virus provided by the National Centre for Foreign Animal Disease (Winnipeg, Manitoba, Canada) (*3*) and an H1 differential PCR (*6*) provided by the National Microbiology Laboratory (Winnipeg, Manitoba, Canada).

We tested 5 herds (herds A–E) in which pandemic (H1N1) 2009 virus was diagnosed (Table). Herds A, B, and D were finishing herds, herd C was a nursery herd, and herd E was a farrowing herd. Herd sizes ranged from 850 to 4,100 pigs. For herds C and D, human illness 16–92 days before the outbreak was reported. The owners of herds A and B reported that they received pigs from a previously infected herd. For persons in contact with herd E, no ill persons were reported, and no pigs from previously affected herds were received before the outbreak. Pigs in herd E were vaccinated for swine influenza A (H1N1) and (H3N2) viruses with an autogenous vaccine.

**Table:** Findings from study of 5 tested swine herds in which pandemic (H1N1) 2009 was diagnosed, Manitoba, Canada, 2009

Herd	Type/size	Vaccine	Human illness	Sample 1	Sample 2	Sample 3
A	Finishing, 2,080	None	No (animal spread)	Day 20, 0/32 positive		
B	Finishing, 3,872	None	No (animal spread)	Day 5, 3/32 positive	Day 19, 0/32 positive	Day 67, 0/31 positive
C	Nursery, 4,100	None	Day –16, manager and family sick	Day 19, 0/32 positive		
D	Finishing, 850	None	Day –92, manager and family sick	Day 10, 0/32 positive		
E	Farrowing, 3,100	Subtypes H1N1 and H3N2 (autogenous)	None reported	Day 10, 7/32 positive	Day 17, 0/32 positive	

Clinical signs in pigs were reported to be mild, with no deaths. However, herd D, co-infected with porcine reproductive and respiratory syndrome virus, *Mycoplasma hyopneumoniae*, and porcine circovirus, reported a 1% outbreak-associated death rate. No vomiting or diarrhea was reported in any pigs infected with the virus.

Nasal swabbing of the pigs demonstrated that pandemic (H1N1) 2009 virus was no longer detected in swine 10–20 days after clinical signs appeared. When tested again the week before slaughter (day 67), herd B showed no evidence of virus shedding.

## Conclusions

We demonstrated that field infections of pandemic (H1N1) 2009 in swine are similar in duration to infections with other swine influenza viruses. In the herds studied, the virus caused mild illness identical to the clinical signs typical of swine influenza (*7*). Sampling by nasal swab indicated that pandemic (H1N1) 2009 virus sheds for up to 20 days after clinical signs appear. Our findings support the laboratory work of Lange et al., who established that pigs experimentally infected with this strain intermittently shed the virus 6–11 days postinfection and ceased excretion by day 11 (*5*). Shedding of the circulating strains of swine influenza in nasal secretions stops by 5–7 days postinfection (*7**–**9*). Our study also indicates that autogenous vaccine prepared with circulating H1N1 subtype may not protect pigs from pandemic (H1N1) 2009 infection.

Our study has several limitations. Other swine viruses, such as porcine reproductive and respiratory syndrome virus, may interfere with the detection of swine influenza viruses from nasal swabs (*8*), and we did not test for other viruses. In addition, the virus can be difficult to diagnose in nursery pigs because of maternal antibodies and low levels of exposure (*8*), which may have affected the samples from the nursery herd. The small sample size and the unknown sensitivity of the PCR in this specific application also limit the findings of our study.

The swine herds we studied quickly cleared the virus after infection. This study supports the recommendations developed by the Canadian Food Inspection Agency (*10*) and the World Organisation for Animal Health (OIE) (*11*). These guidelines state that pigs infected with pandemic (H1N1) 2009 virus should be managed similarly to herds infected with any swine influenza virus. On the basis of our study findings, restrictions of trade or slaughter of pigs as a public health intervention are irrational actions.

Only 10 countries have reported pandemic (H1N1) 2009 infection in commercial swine to the OIE (*12*). Whether pandemic (H1N1) 2009 will become established in swine populations worldwide remains to be seen. All countries should implement vigilant surveillance for, and monitor for changes in the structure and behavior of, the virus.

## References

[1] Centers for Disease Control and Prevention. Swine influenza A (H1N1) infection in two children—southern California, March–April 2009. MMWR Morb Mortal Wkly Rep. 2009;58:400–2. 19390508

[2] World Health Organization. Global alert and response. Current WHO phase of pandemic alert [cited 29 Jan 2010]. http://www.who.int/csr/disease/avian_influenza/phase/en

[3] Novel Swine-Origin Influenza A (H1N1) Virus Investigation Team, Dawood FS, Jain S, Finelli L, Shaw MW, Lindstrom S, et al. Emergence of a novel swine-origin influenza A (H1N1) virus in humans. N Engl J Med. 2009;360:2605–15. 10.1056/NEJMoa0903810 19423869

[4] Howden KJ, Brockhoff EJ, Caya FD, McLeod LJ, Lavoie M, Ing JD, An investigation into human pandemic influenza virus (H1N1) 2009 on an Alberta swine farm. Can Vet J. 2009;50:1153–61. 20119537 PMC2764467

[5] Lange E, Kalthoff D, Blohm U, Teifke JP, Breithaupt A, Maresch C, Pathogenesis and transmission of the novel swine-origin influenza virus A/H1N1 after experimental infection of pigs. J Gen Virol. 2009;90:2119–23. 10.1099/vir.0.014480-0 19592456

[6] Leblanc JJ, Li Y, Bastien N, Forward KR, Davidson RJ, Hatchette TF. Switching gears for an influenza pandemic: validation of a duplex RT-PCR for simultaneous detection and confirmation of pandemic (H1N1) 2009. J Clin Microbiol. 2009;47:3805–13. 10.1128/JCM.01344-09 19794033 PMC2786647

[7] Olsen CW, Brown IH, Easterday BC, Van Reeth K. Swine influenza. In: Straw BE, Zimmerman JJ, D’Allaire S, Taylor DJ, editors. Diseases of swine. 9th ed. Ames (IA): Blackwell Publishing; 2006. p. 469–82.

[8] Gillespie TG. Diagnosing endemic swine influenza virus in nursery pigs using cross-sectional serologic profiling. Swine Health and Production. 1999;7:81–3.

[9] Janke BH. Diagnosis of swine influenza. Swine Health and Production. 2000;8:79–84.

[10] Canadian Food Inspection Agency. News release. Management of pandemic H1N1 in swine herds [cited 2009 Oct 5]. http://www.inspection.gc.ca/english/corpaffr/newcom/2009/20090724e.shtml

[11] World Organisation for Animal Health. Press releases. Pandemic (H1N1) 2009: the OIE reiterates its recommendations to animal health authorities worldwide [cited 2009 Oct 5]. http://www.oie.int/eng/press/en_090713.htm

[12] World Organisation for Animal Health. WAHID interface. Weekly disease information [cited 2009 Dec 3]. http://www.oie.int/wahis/public.php?page=weekly_report_index&admin=0

